# Supplementary figures and images for: Matching plasma and tissue miRNA expression analysis to detect viable ovarian germ cell tumors
Source: PLoS One. 2025 May 9;20(5):e0322477. doi: 10.1371/journal.pone.0322477 (PMC12063854; doi:10.1371/journal.pone.0322477)

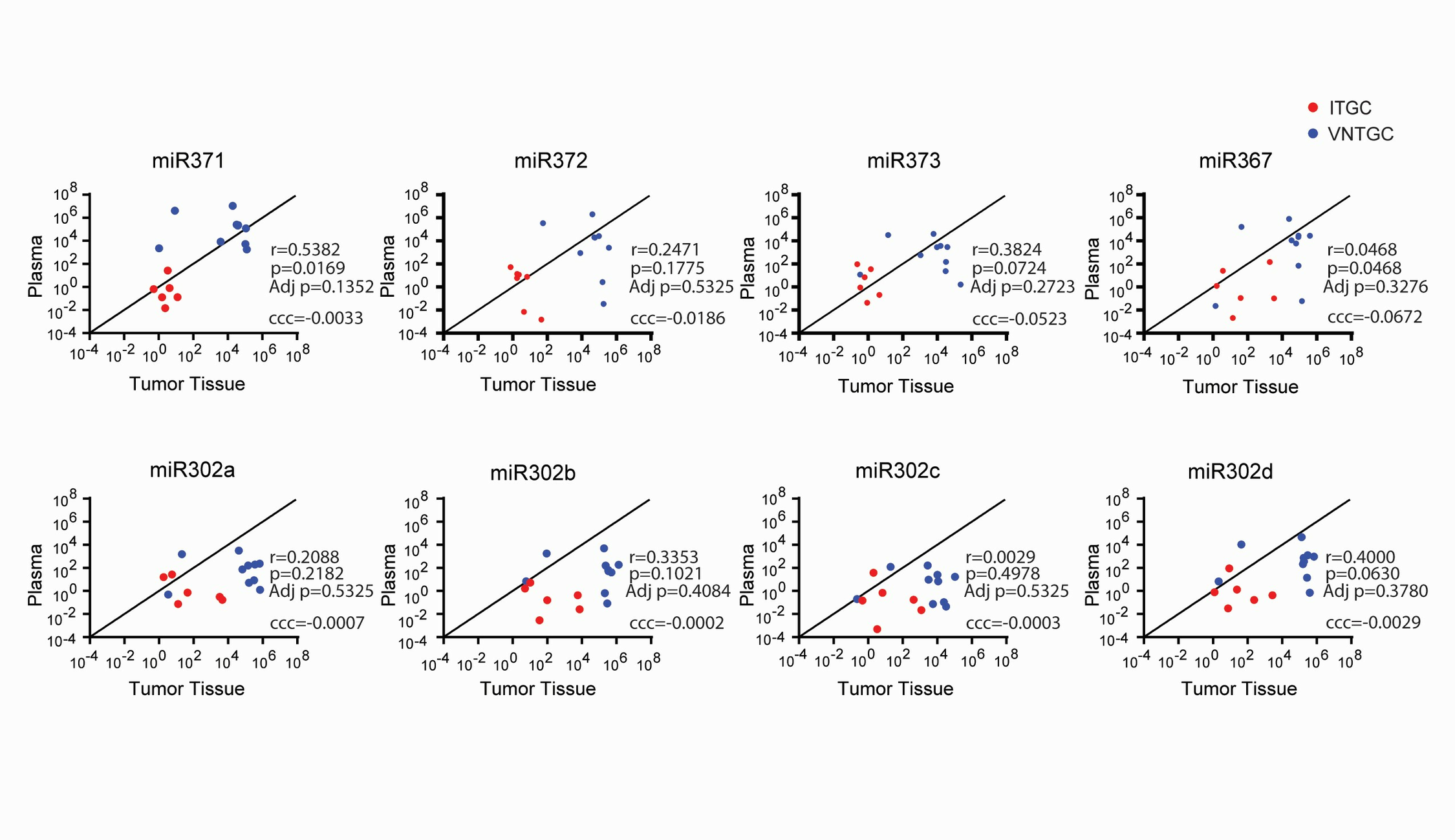

Supplement: S1 Fig — Red dots represent patients with ITGC tumors, and blue dots represent VNTGC tumors. The Correlation Coefficient (r) was calculated in Graphpad Prism, and the Concordance Correlation Coefficient (CCC) was determined as described in statistical analysis section. (TIF) [file pone.0322477.s001.tif]

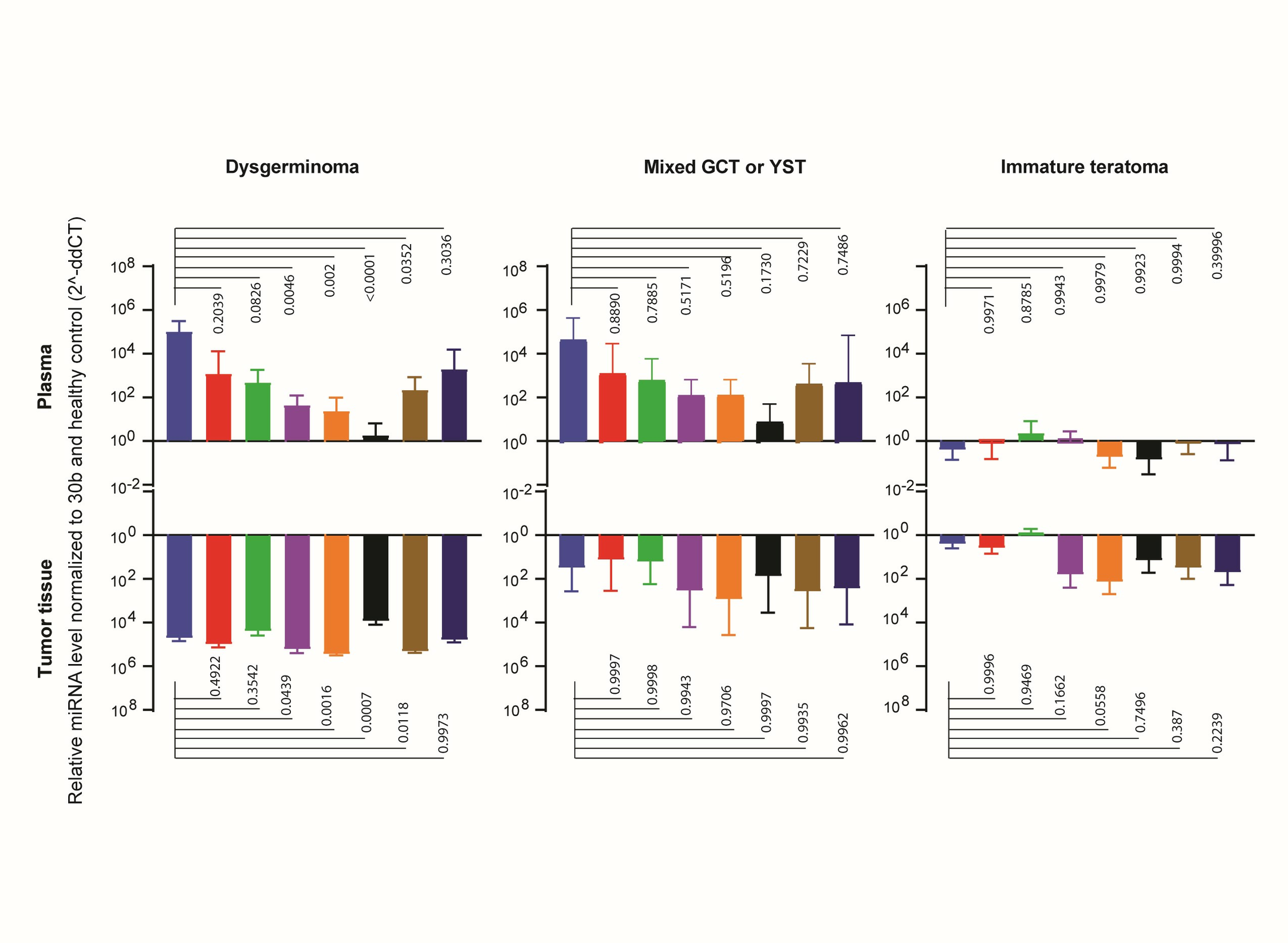

Supplement: S2 Fig — Bar graphs represent relative miRNA expression in teratoma, dysgerminoma, and mixed GCT in both plasma (top) and tumor tissue (bottom). Statistical comparisons were performed using one-way ANOVA followed by Dunnett’s post-hoc test to identify differential expression of miRNAs in specific tumor types. (TIF) [file pone.0322477.s002.tif]

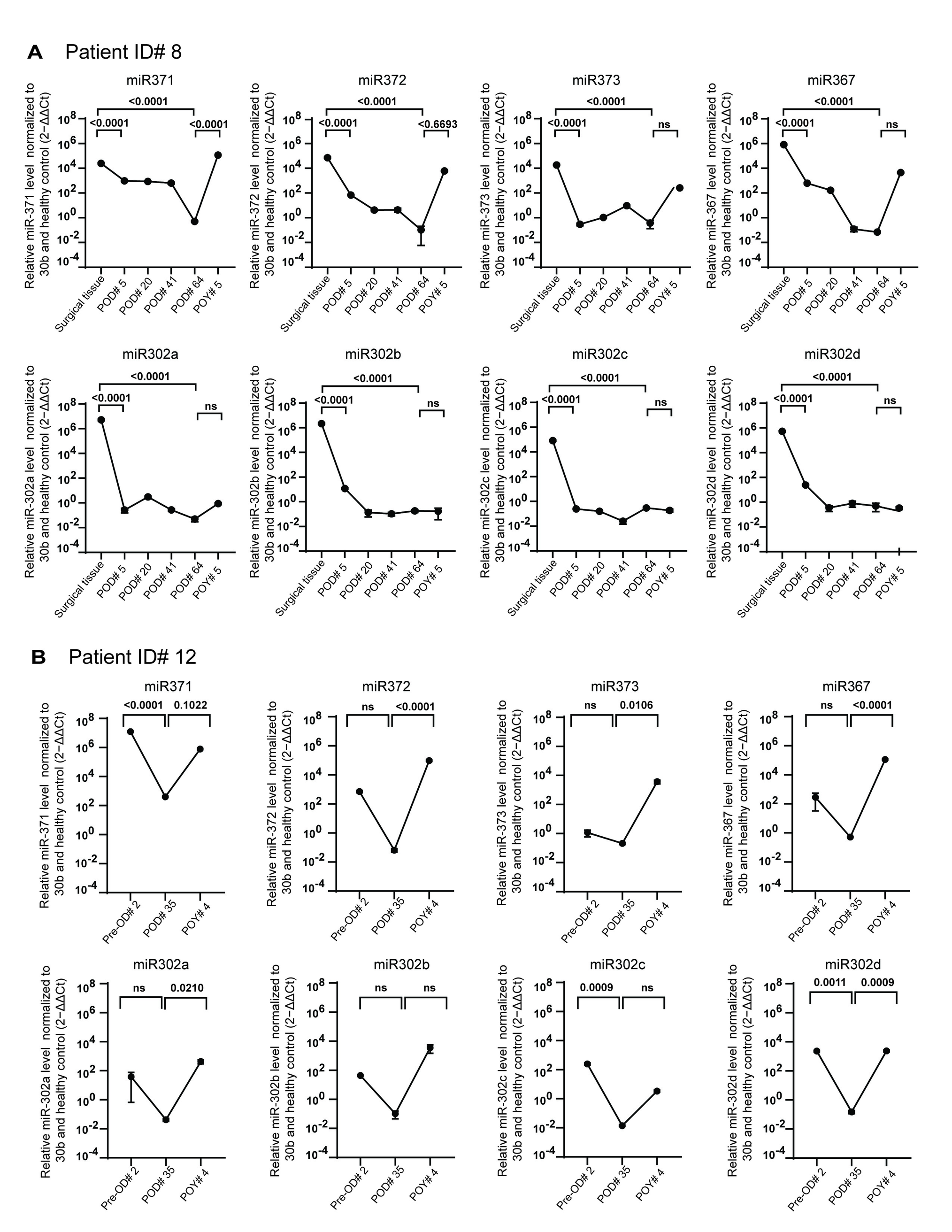

Supplement: S3 Fig — Statistical differences were analyzed using a one-way ANOVA test. Each graph represents data from an individual patient with bars indicating the mean of three technical replicates. (TIF) [file pone.0322477.s003.tif]
