# Supplementary material for: Matching plasma and tissue miRNA expression analysis to detect viable ovarian germ cell tumors
Source: PLoS One. 2025 May 9;20(5):e0322477. doi: 10.1371/journal.pone.0322477 (PMC12063854; doi:10.1371/journal.pone.0322477)
Supplement: Attachment — Submitted filename: rebuttal letter-final.docx [file pone.0322477.s005.docx]

Dear Dr. Decock,

We sincerely thank you and the reviewers for the thoughtful comments and constructive feedbacks on our manuscript titled “Matching plasma and tissue miRNA expression analysis to detect viable ovarian germ cell tumors.” We appreciate the opportunity to revise our manuscript for potential publication in *PLOS ONE*.

We have carefully addressed all the points raised by the reviewers and academic editor. Below, we provide a point-by-point response to each comment, followed by details of the changes made to the manuscript. We believe that the revisions have strengthened the manuscript significantly.

**Reviewer #1**

**Introduction**
**1. Please define at least once the acronyms used in the introduction.**

- **Response:** All acronyms, including FIGO, OGCT, and miRNAs, have now been defined at their first mention in the Introduction.

(OGCTs ) defined in Page 5 line 75

viable non-teratoma germ cell (VNTGC): Page 5 line 75

immature teratoma germ cell (ITGC): Page 5 line 77

germ cell tumor (GCTs): Page 5 line 82

**Methods**
**2. Line 140: Provide the manufacturer and catalog number for the Streck tubes used in the study.**

- **Response:** We have included the manufacturer (Streck, Cat#: 230470) in the Methods section. Page 8 line 143

**3. Line 141: Instead of using the term ‘in a standard fashion,’ please provide the exact conditions for the centrifugation.**

- **Response:** The specific conditions for centrifugation have now been added.

It is changed to ‘’ was centrifuged at 1,600 x g for 15 minutes at room temperature. The top layer was transferred to a new tube and centrifuged at 14,400 x g for 10 minutes at room temperature’’.Page 8 line 143

**Results**
**4. Line 204: Please explain the definition of FIGO and include a reference.**

- **Response:** We have included the explanation as follows. “Most patients (61%) had International Federation of Gynecology and Obstetrics (FIGO) stage I disease where the primary tumor is limited to the ovaries or fallopian tubes at initial diagnosis.’’ Page 11 line 219-220

**5. Table 1: Please include the definitions of the acronyms at the bottom of the table for better interpretation.**

- **Response:** All acronyms in Table 1 are already defined in the bottom of the table.

**6. Figure 3: Instead of showing the results in graphs for visual comparison, consider plotting correlation graphs to reach miRNA so that this correlation can be quantifiable.**

- **Response:** Thank you for your valuable suggestion. We have created a supplementary (Fig.S1) figure that includes separate correlation graphs for each miRNA, illustrating the relationship between miRNA levels in plasma and tumor tissue. Additionally, we have calculated the concordance correlation coefficient (CCC) for each graph to provide a quantifiable measure of correlation.

**7. Figure 4: Have you done statistical analysis comparing the time points assessed? That would be important to show in the graph. Especially the comparison of the first point (Surgical tissue, Pre-OD# 2) to the other time points.**

- **Response:** Thank you for your insightful suggestion. To assess whether the changes in miRNA expression at different timepoints were statistically significant, we conducted an ANOVA test for each miRNA. The results, along with separate graphs for each miRNA, have been included in a supplementary figure (see supplementary Fig 3) to provide a clear and comprehensive representation of the statistical significance of the observed trends. Since each graph in Figure 4 represents an individual patient, we performed the statistical analysis using technical replicates. The statistical analysis are explained in page 11, line 206-208..

**Reviewer #2**

1. **Lack of data showing relation to mRNA targets of miR-371-3/302/367.** The evidence of these miRNAs presence and overexpression in OGCT patients, decrease after surgery, and re-increase well-after resection is very well-established in the paper. However, miRNAs do not act alone to cause oncogenicity, and therefore, it would be further solidifying of the authors’ data to show inverse patterns of known targets of these miRNA such as LATS2, TGFBR2, CDKN1A, or PIK3R1 in each of the patients shown in Figures 4 and 5. Although the authors’ intention was not to provide evidence for a putative pathway, it would certainly add to the significance of their findings. To even further concretize the authors' findings, they could create a ceRNA network related to all miRNA in the paper to show possible avenues of action instead of simple correlations.

- **Response:** We appreciate the reviewer’s thoughtful input. Unfortunately, most of the tissue samples have been exhausted, preventing further analysis of downstream targets. As mentioned by the reviewer, the primary objective of this study is to confirm over-expression of these micro-RNA clusters also in ovarian germ cell tumors and that they can be detected in the plasma of patients with clinical evidence of non-teratoma germ cell tumors. These data could be therefore used to inform future studies that will confirm the diagnostic utility of circulating miRNAs in OGCT to help guiding clinical decision making in the postoperative setting and on surveillance.

**2. Data-driven comparison of OGCT to TGCT.**

a. The authors’ rely on the similarity of testicular germ cell tumors within the introduction, and discussion, sections but do not provide any comparative data. Considering that the TGCT are the more well-established tumor to miRNA dysregulation, a comparison to currently available data from these tumors would further solidify the connection between these primordial sex cells. Additionally, it could provide avenues for further investigation between the two to find similarities, differences, and correlations.

- **Response:** The current study has been inspired by the extensive data available in testicular germ cell tumors. Since there are many similarities between ovarian and testicular germ cell tumors in terms of treatments and outcomes, we wanted to explore if the same microRNAs were expressed also in OGCT, and if they were detectable in the peripheral blood. The corresponding author and many co-authors on this manuscript are involved in the clinical utility validation of miR371 in testicular germ cell tumors, leading large prospective studies (SWOG S1823). d. The largest and most clinically relevant studies in testicular germ cell tumors have been cited throughout the manuscript and discussed in the” discussion” paragraph. Our primary objective was not to compare OGCT with testicular GCT; it was to test whether or not these microRNAs were also expressed by OGCT and detectable in the peripheral blood. Any comparison between the OGCT and testicular GCT will be highly inaccurate and underpowered because of:

the heterogeneous patients’ populations. These miRNAs expression largely depend on clinical stage and tumor histology (not expressed in teratoma, lower expression in seminoma than in non seminoma). A comparison should be done by choosing matching testis cancer cases by stage, tumor subtypes and timing of blood collection (ex. prior to chemotherapy or surgery; post chemotherapy or surgery, etc..). This is unfortunately not feasible. Moreover, there will be no statistical power considering the very exiguous number of cases in each subgroup of the OGCT

**3. Addressing tumor heterogeneity.** a. Within the authors’ cohort, there is a wide-range of expression levels of mentioned miRNAs between patients (Figure 3). It would be helpful to delineate in a supplementary and separate figure which miRNAs are most overrepresented in specific tumor types as addressed in the discussion section. It’s one thing to recognize the difference in expression between tumor types, but it’s another, more useful, thing to present those differences quantitatively in graphical form. This could also add to the conclusion that germ cell tumors express specific miRNAs, regardless of sex type.

- **Response:** Thank you for your insightful comment regarding tumor heterogeneity and the variation in miRNA expression across patients. We acknowledge the wide range of expression levels observed in our cohort (Figure 3) and appreciate the suggestion to present these differences quantitatively for each tumor type. To address this, we have generated a supplementary figure (Fig S2) that provides a clearer quantitative comparison, of different miRNAs expression levels in different tumor types. Additionally, we have distinguished the tumor types using different colors in Figure 3 to enhance visualization.

### Reviewer #3

**1. LDH levels: Specify whether they are higher or lower than normal.**

- **Response:** We clarified in the LDH level by changing “abnormal LDH” to “ above ULN” in Page 11 lines 225, 226
- **2. Line 212: Verify AFP data.**
- **Response:** We verified the data and corrected the number in the text to 21, consistent with the table. Page 11 line 228

**3. Use higher-resolution images for Figures 1 and 2.**

- **Response:** Figures 1 and 2 have been replaced with higher-resolution versions to improve readability.

**4. Clearly reference figure numbers and sub-figures in the Results section.**

- **Response:** Figure and sub-figure numbers have been explicitly referenced throughout the Results section for clarity.

**5. Conclusion should specifically point out the limitations of this study**

- **Response:** We changed the last sentence of conclusion as follow, to point out the limitation. “Although these findings are based on a limited retrospective patient cohort, they suggest a potential role for miRNAs them as clinical biomarkers in OGCT”. Page 19 line 397-399.
